# CapsEnhancer: An Effective Computational Framework for Identifying Enhancers Based on Chaos Game Representation and Capsule Network

**DOI:** 10.1021/acs.jcim.4c00546

**Published:** 2024-07-01

**Authors:** Lantian Yao, Peilin Xie, Jiahui Guan, Chia-Ru Chung, Yixian Huang, Yuxuan Pang, Huacong Wu, Ying-Chih Chiang, Tzong-Yi Lee

**Affiliations:** † Kobilka Institute of Innovative Drug Discovery, School of Medicine, The Chinese University of Hong Kong, Shenzhen 518172, China; ‡ School of Science and Engineering, The Chinese University of Hong Kong, Shenzhen 518172, China; § School of Medicine, The Chinese University of Hong Kong, Shenzhen 518172, China; ∥ Department of Computer Science and Information Engineering, National Central University, Taoyuan 320317, Taiwan; ⊥ Division of Health Medical Intelligence, Human Genome Center, The Institute of Medical Science, The University of Tokyo, Tokyo 108-8639, Japan; # Institute of Bioinformatics and Systems Biology, National Yang Ming Chiao Tung University, Hsinchu 300093, Taiwan; ¶ Center for Intelligent Drug Systems and Smart Bio-devices (IDS2B), National Yang Ming Chiao Tung University, Hsinchu 300093, Taiwan

## Abstract

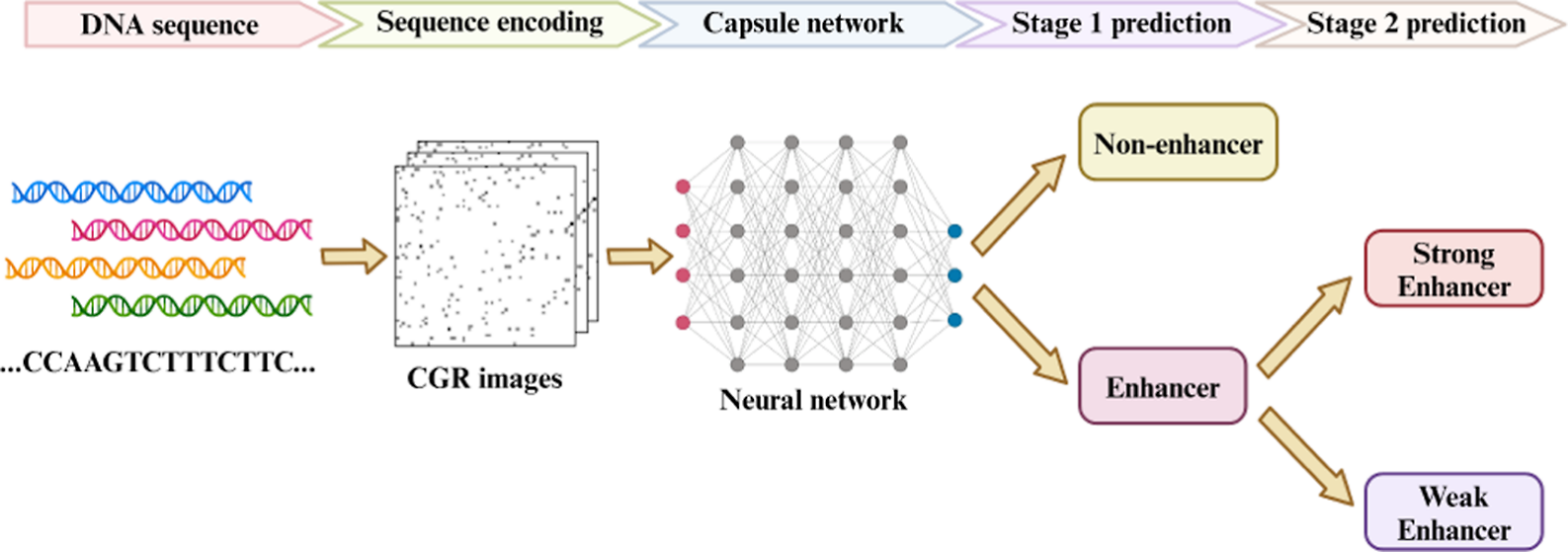

Enhancers are a class of noncoding DNA, serving as crucial regulatory elements in governing gene expression by binding to transcription factors. The identification of enhancers holds paramount importance in the field of biology. However, traditional experimental methods for enhancer identification demand substantial human and material resources. Consequently, there is a growing interest in employing computational methods for enhancer prediction. In this study, we propose a two-stage framework based on deep learning, termed CapsEnhancer, for the identification of enhancers and their strengths. CapsEnhancer utilizes chaos game representation to encode DNA sequences into unique images and employs a capsule network to extract local and global features from sequence “images”. Experimental results demonstrate that CapsEnhancer achieves state-of-the-art performance in both stages. In the first and second stages, the accuracy surpasses the previous best methods by 8 and 3.5%, reaching accuracies of 94.5 and 95%, respectively. Notably, this study represents the pioneering application of computer vision methods to enhancer identification tasks. Our work not only contributes novel insights to enhancer identification but also provides a fresh perspective for other biological sequence analysis tasks.

## Introduction

Enhancers, short noncoding DNA sequences interspersed throughout the genome, play an indispensable role in the orchestration of gene expression and, by extension, every biological process in living organisms.^1−3^ These unique genomic elements are known to amplify the transcription rate of their associated genes, acting as regulators in the vast genomic machinery. Enhancers facilitate the binding of proteins, such as transcription factors and coactivators, which modulate transcription initiation, thereby influencing many cellular activities such as differentiation, development, and responses to environmental stimuli.^4^ Notably, enhancers can function from variable distances away from the genes they regulate and can be located upstream, downstream, or even within intronic regions of these genes. The flexible nature of their operation within the genomic landscape enhances their regulatory capacity, making them vital components in the architecture of life.^5−7^

The functional importance of enhancers extends beyond merely amplifying gene expression; they are critical in determining the spatiotemporal patterns of gene activity, thereby shaping the identity and function of each cell type. In essence, enhancers are at the heart of cellular diversity and organismal complexity. Their malfunctioning is associated with various genetic disorders, including cancer, highlighting their importance in maintaining cellular homeostasis.^8^ Traditional enhancer identification methods,^9−11^ such as ChIP-seq, although capable of identifying enhancers, also face challenges, including high costs, low throughput, and extensive starting material requirements. These challenges make them time-consuming, labor-intensive, and expensive. Moreover, the vastness and complexity of the human genome make the large-scale application of these experimental methods impractical.^12^ As a result, the scientific community is increasingly utilizing computational methodologies to identify and classify enhancers. The advent of next-generation sequencing technologies has led to the development of numerous computational strategies aimed at distinguishing enhancers from other noncoding genomic regions, offering an economical and efficient alternative to traditional experimental approaches.^13^

Computational methods are generally classified into two categories: based on traditional machine learning classifiers and deep learning. Support vector machine (SVM) and random forest (RF) algorithms are frequently employed for enhancer classification. Liu et al. proposed a method called iEnhancer-2L, integrating the pseudo k-tuple nucleotide composition (PseKNC) of DNA sequences and utilizing SVM for enhancer identification.^14^ Jia et al. introduced a tool named EnhancerPred, integrating biprofile bayes (BPB), nucleotide composition (NC), and PseNC, constructing a classifier using SVM.^15^ Lim et al. developed a RF-based tool called iEnhancer-RF, integrating six kinds of features of DNA sequences.^16^ Methods based on ensemble learning are also employed for the recognition of enhancers. In 2018, Liu et al. proposed an ensemble learning method named iEnhancer-EL, utilizing PseKNC, Kmer, and subsequence profile of sequences to predict enhancers.^17^ Similarly, Wang et al. developed Enhancer-FRL, integrating ten kinds of features and employing five machine learning methods, including SVM and RF, to predict enhancers and their activities.^18^ Gill et al. developed a deep forest-based tool, NEPERS, integrating four kinds of features to identify enhancers.^19^

In recent years, in addition to traditional machine learning algorithms, due to the development of deep learning, an increasing number of enhancer classifiers based on deep learning have emerged. Nguyen et al. developed iEnhancer-ECNN, utilizing one-hot encoding of sequences and convolutional neural network (CNN) to predict enhancers.^20^ Le et al. introduced BERT-Enhancer, utilizing a BERT pretraining model to extract sequence encoding, followed by CNN to build a classifier.^21^ Niu et al. developed a tool named iEnhancer-EBLSTM, using Kmers information from DNA sequences and employing bidirectional LSTM (BiLSTM) to construct an enhancer classifier.^22^ A summary of relevant work is presented in Table 1.

**Table 1:** Summary of Existing Tools for Enhancer Identification

no.	method	feature encoding	algorithm	year	reference
1	iEnhancer-2L	PseKNC	SVM	2016	(14)
2	EnhancerPred	BPB, NC, PseNC	SVM	2016	(15)
3	iEnhancer-EL	Kmer, subsequence profile, PseKNC	ensemble learning	2018	(17)
4	iEnhancer-ECNN	one-hot encoding, Kmers	CNN	2019	(20)
5	iEnhancer-XG	k-spectrum profile, mismatch k-tuple, subsequence profile, PSSM, PseDNCb	XGBoost	2021	(23)
6	BERT-Enhancer	BERT encoding	CNN	2021	(21)
7	iEnhancer-EBLSTM	Kmers	BiLSTM	2021	(22)
8	iEnhancer-RF	NBP, DBP, ANF, NCP, ENAC, XY K-GAP	RF	2021	(16)
9	iEnhancer-RD	Kmers, PseKNC, KPCV	DNN	2021	(24)
10	spEnhancer	Kmers	BiLSTM	2021	(25)
11	Enhancer-FRL	ANF, CKSNAP, DAC, ENAC, Kmers, NCP,PseTIIP, SCPseDNC, SCPseTNC, TACC	SVM, RF, KNN, naive Bayesian, LightGBM	2022	(18)
12	iEnhancer-BERT	BERT encoding	CNN	2022	(26)
13	iEnhancer-ELM	Kmer, BERT encoding	MLP	2023	(27)
14	iEnhancer-DCSA	Word2vec	dual-scale CNN, spatial attention	2023	(28)
15	iEnhancer-SKNN	Kmer, PseDNC, PCPseDNC and Z-Curve9	ensemble learning	2023	(29)
16	NEPERS	PSTNPss, PSTNPdss, CKSNAP, NCP	deep forest	2023	(19)

While the computational methodologies mentioned above exhibit promising outcomes, with each demonstrating distinct merits, additional investigation is warranted for the following reasons. Prior studies have demonstrated the discriminative capabilities of utilizing Kmers in distinguishing biosequences with diverse functionalities.^30−32^ For example, Li et al. proposed a method called GCR-Net, which models Kmers of genomic sequences hierarchically to enhance the prediction of translation initiation sites.^33^ Nevertheless, extant models, including CNNs, exhibit suboptimal performance in effectively learning the intricate associations between Kmers and their respective frequencies. An efficacious method for sequence encoding, chaos game representation (CGR), has the capability to transform biosequences into two-dimensional images.^34^ By encoding Kmers frequencies as visual representations, employing computer vision methods to discern and acquire patterns inherent in CGR-encoded images is straightforward.^35^ Moreover, although CNNs have achieved a series of successes in recent years, they are not without limitations. For instance, CNNs lack an understanding of the hierarchical structure of objects. Traditional CNNs struggle to capture the hierarchical structure and part-whole relationships of objects, leading to limitations in comprehending the relationship between the overall and local features of objects.^36,37^ In recent years, researchers have been continuously developing novel methods to address the aforementioned limitations and applying them to various tasks. Guo et al. proposed a variational gated autoencoder-based feature extraction model to extract complex contextual features and infer disease-miRNA associations.^38^ Additionally, a method called MCANet, which integrates multiscale convolution and self-attention mechanisms, adaptively reveals spatial-temporal contextual dependence to enhance Poly(A) signal prediction.^39^ Wang et al. introduced a cross-feature enhancement module, which effectively reduces information redundancy and facilitates the integration and modeling of complementary features using attention mechanisms.^40^

To further address constraints of CNNs, a new generation of neural networks, known as capsule networks, has emerged.^41^ Capsule networks introduce the concept of capsules to better capture the spatial hierarchical structure within objects. Each capsule represents a specific entity or part, and the relationships between capsules can be modeled. This contributes to an enhanced understanding of object hierarchical structures by the network. In recent years, capsule networks have been applied to tasks in bioinformatics, demonstrating satisfactory performance.^42−45^

**Figure 1:**
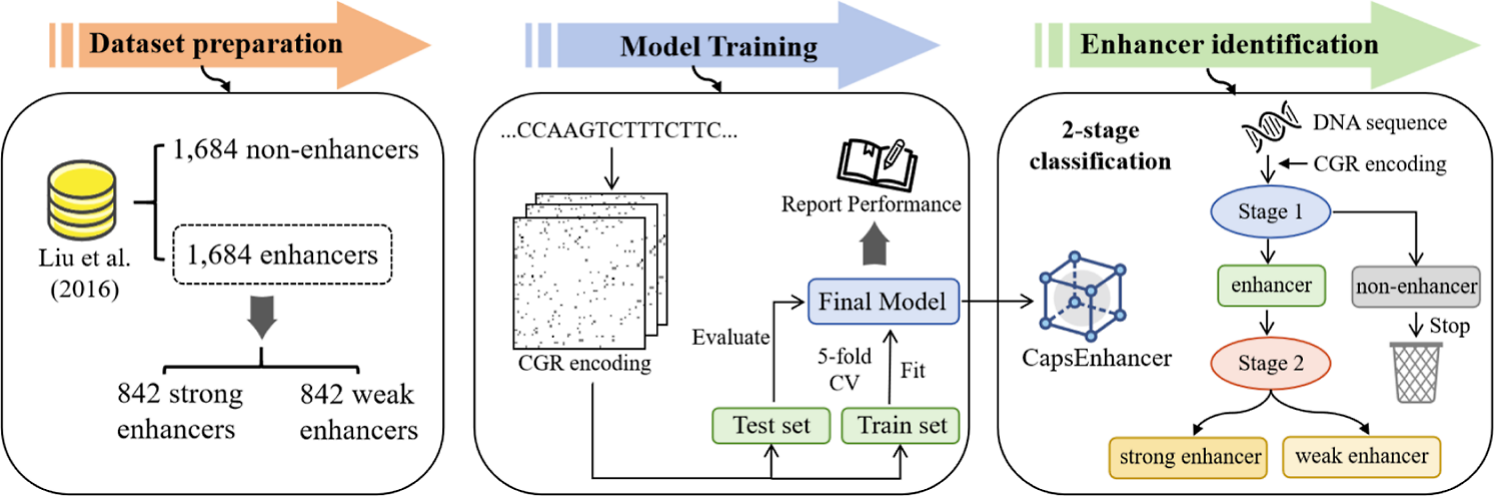
Workflow of CapsEnhancer. First, we utilized benchmark data sets from previous studies. Subsequently, each DNA sequence was encoded using CGR encoding and represented as corresponding two-dimensional images. The model was then constructed using an architecture based on capsule networks. Hyperparameter adjustment was performed through fivefold cross-validation, and the model was evaluated using an independent test set, with the subsequent reporting of model performance metrics. The trained model was ultimately employed for enhancer identification, constituting a two-stage task. The first stage focused on discerning enhancers from nonenhancers, while the second stage aimed to predict enhancer strength, i.e., strong enhancers versus weak enhancers. The second stage employs the same FCGR images as the first stage to maintain consistency in the input representation. Capsule networks are used in both stages to build the models.

In this study, we proposed a new scheme named CapsEnhancer, designed to achieve the identification of enhancers and their strength. The workflow of CapsEnhancer is shown in Figure 1. Experimental results demonstrate that CapsEnhancer achieves satisfactory performance on benchmark data sets. The main contributions of this study can be summarized as follows.

(1) We designed a two-stage computational framework called CapsEnhancer to identify enhancers and their strengths. The first stage of CapsEnhancer focuses on enhancer recognition, distinguishing between enhancer and nonenhancer. The second stage involves predicting enhancer strength, specifically discerning between strong and weak enhancers.
(2) CapsEnhancer uses CGR encoding to represent each DNA sequence as an image. Through this encoding method, it can effectively represent Kmers and their frequencies.
(3) CapsEnhancer employs a capsule network-based architecture to learn local and global features from the “images” transformed from DNA sequences. CapsEnhancer represents the pioneering adoption of computer vision strategies for enhancer identification.
(4) Experimental results demonstrate that CapsEnhancer attains state-of-the-art performance in the two-stage task. In comparison to previous methods, CapsEnhancer exhibits significant improvements, achieving an 8% increase in accuracy during the first stage and a 3.5% improvement in the second stage. Beyond providing a robust solution for enhancer identification, our framework introduces a novel perspective for other biological sequence analysis tasks.

## Materials and Methods

### Benchmark Data Set

In order to facilitate fair comparisons, we employed the data set constructed by Liu et al.,^14,17^ which has been widely used in enhancer prediction tasks.^15,16,18,20,29^ The enhancers within this data set were derived from nine distinct cell lines, wherein they were isolated as DNA sequences from short 200 bp clips of uniform length. Subsequently, the CD-HIT software was employed to eliminate paired sequences exhibiting a similarity surpassing 20%.

The final data set can be represented as follows
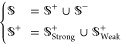
1where the subset  comprises 1484 enhancer samples, and  comprises 1484 nonenhancer samples, forming the first stage of the data set. In addition,  consists of 742 strong enhancer samples, and  comprises 742 weak enhancer samples, constituting the second stage of the data set. The independent test set is utilized for assessing the model’s performance, and it is sourced from the work of Liu et al., encompassing 100 strong enhancers, 100 weak enhancers, and 200 nonenhancers.

To facilitate a more comprehensive understanding of the distinctions between positive and negative samples, the GC content of data sets for two distinct stages was plotted, as presented in Figure S1. It is evident that enhancers exhibit a higher GC content compared to nonenhancers. Furthermore, strong enhancers also display a higher GC content when contrasted with weak enhancers.

### Architecture Overview of CapsEnhancer

**Figure 2:**
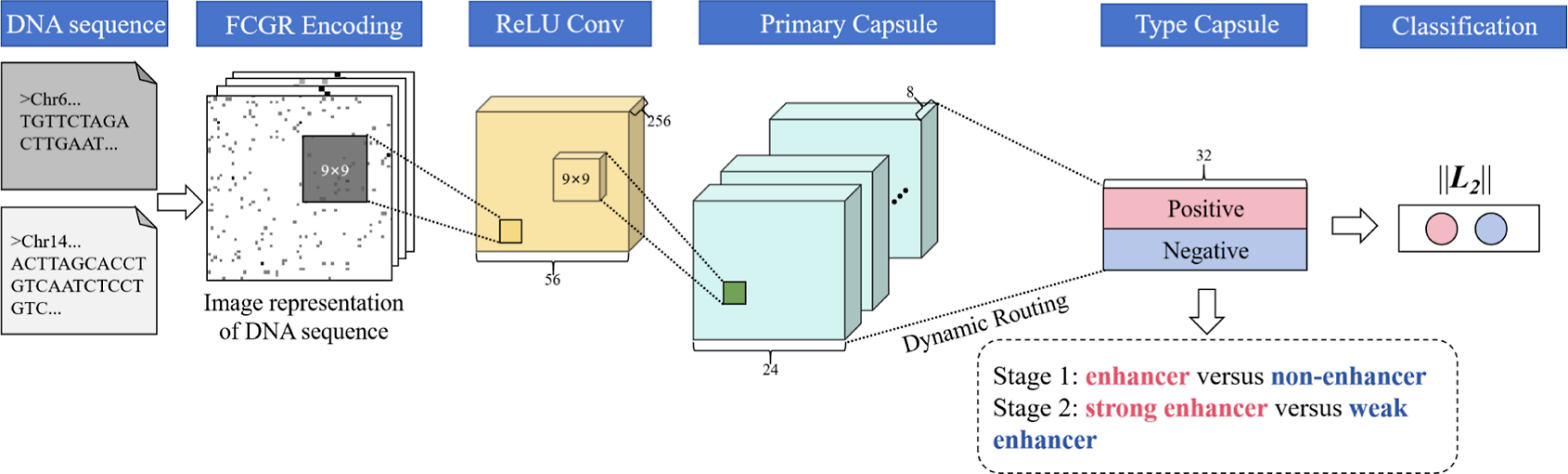
Architecture of CapsEnhancer. First, DNA sequences are encoded using CGR encoding and represented as 2D images. Subsequently, they are input into a Conv2D for preliminary feature extraction. Following this, the data is fed into a capsule network, which consists of a primary capsule layer and a type capsule layer. The primary capsule layer includes a Conv2D for further extracting local features. Then, a dynamic routing algorithm is utilized to capture the spatial relationships of features, resulting in the type capsule layer. As the task is a standard binary classification, the type capsule layer comprises two capsules, corresponding to the positive class and the negative class (stage 1: enhancer versus nonenhancer; stage 2: strong enhancer versus weak enhancer). Finally, the prediction probabilities for the two classes are obtained by calculating the lengths of the capsules in the type capsule layer.

The model architecture of CapsEnhancer is illustrated in Figure 2. CapsEnhancer is a two-stage framework wherein the first stage aims to identify enhancer and nonenhancer, while the second stage focuses on distinguishing between strong enhancer and weak enhancer categories. Initially, DNA sequences are transformed into images through CGR encoding. Subsequently, a 2-dimensional convolutional neural network (Conv2D) is employed to preliminarily extract features from these images. The acquired preliminary features are then fed into a capsule network for further feature extraction and spatial modeling from the images. The main notations of this study are summarized in Table 2.

**Table 2:** Main Notations and Descriptions

notation	description
*N*	the size of the FCGR images
*m*	dimension of each primary capsule
*n*	dimension of each type capsule
*u*_*i*_	primary capsules
*V*_*j*_	type capsules
*W*	weight matrix in capsule networks
*c*_*i*,*j*_	coupling coefficients
*p*	prediction probability

### CGR Encoding

CGR is a mathematical method that employs iterated function systems to convert sequential data into a fractal depiction within a two-dimensional space. CGR is a milestone in graphical bioinformatics and is considered a powerful tool for feature encoding in biological sequences, including DNA, RNA, and protein sequences.^35,46^

**Figure 3:**
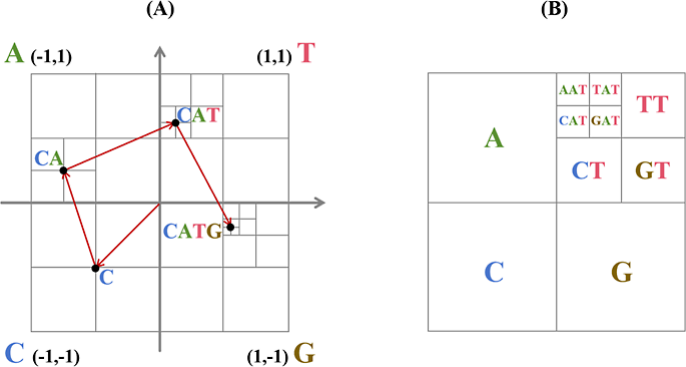
(A) Applying CGR encoding to an example sequence: CATG. (B) Dividing the CGR space during the iterative process.

We employed CGR encoding to encode DNA sequences in this study. Initially, allocate the four nucleotides (A, C, G, T) to the four vertices of a square. Figure 3A provides an illustrative example of encoding a sequence using CGR representation. For a DNA sequence *s* of length *n*, where *s* = *s*_1_, ..., *s*_*i*_, ..., *s*_*n*_ and *s*_*i*_ ∈ {A, C, G, T}, the coordinates of the new nucleotide *s*_*i*_ in the sequence are determined by the current amino acid type and the coordinates of the preceding nucleotide *s*_*i*–1_. The position of *s*_*i*_ is located halfway along the line connecting the current position and the vertex associated with the nucleotide. CGR encoding of sequence *s* is a two-dimensional representation of ordered pairs (*x*_1_, *y*_1_) through (*x*_*i*_, *y*_*i*_) to (*x*_*n*_, *y*_*n*_), where (*x*_*i*_, *y*_*i*_) is defined as follows
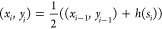
2where (*x*_0_, *y*_0_) = (0, 0) and
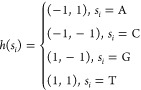
3

Figure 3B illustrates the partitioning of the CGR space during the iterative process. Each subsquare within the CGR space holds distinctive significance. Upon dividing the CGR into four quadrants, the upper right corner encompasses points that symbolize subsequences terminating with the nucleotide T. This is attributed to the fact that the midpoint between any other point within the square and the corner T invariably resides within this quadrant. Upon subdividing this quadrant into four squares in a clockwise order, they, respectively, denote subsequences concluding with TT, GT, CT, and AT. This configuration facilitates the computation of 2-mer counts by tallying the points within these designated subsquares.

In contrast to the precise coordinate representation employed by the original CGR, a discretization method known as the frequency chaos game representation (FCGR) has been introduced to provide a coarser and less susceptible-to-noise abstraction for sequences. FCGR, an extension of CGR, involves a grid-based counting approach for determining the points within the CGR. The initial step of FCGR involves partitioning the CGR image into *N* × *N* regions. Subsequently, the point count within each region serves as the region’s frequency, enabling the compression of the CGR and resulting in an FCGR matrix with dimensions *N* × *N* applicable to input sequences of varying lengths. Therefore, the predefined grid values can serve as a representation of the frequency of Kmers. In this study, we opt for *N* = 64 as the parameter for generating  images corresponding to each DNA sequence.

Figure S2A,B, respectively, depict the FCGR images of an enhancer sequence and a nonenhancer sequence. Their FCGR images exhibit markedly distinct patterns, including highlighted regions in red and blue, which could potentially serve as discriminative signals for distinguishing between enhancer and nonenhancer sequences. These discriminative regions can be spatially modeled by the unique architecture of capsule networks, allowing the learning of their correlations to enhance classification accuracy.

### Capsule Network

Over recent years, CNNs have surpassed numerous conventional models reliant on curated feature extraction, making substantial progress in various domains, including computer vision and bioinformatics. However, CNNs are constrained by their incapacity to comprehend spatial relationships between features and the loss of invariance due to pooling operations. Sabour et al. introduced a novel deep learning paradigm known as capsule network (CapsNet) to circumvent these limitations.^41^

Within the primary capsule layer resides a Conv2D layer, employed for further feature extraction. The outputs from this Conv2D layer are transformed into multiple *m*-dimensional vectors (the dimensionality *m* being a hyperparameter). These *m*-dimensional vectors undergo a nonlinear “squash” function that retains the direction of the vector while constraining its magnitude to a range between 0 and 1.
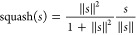
4

**Figure 4:**
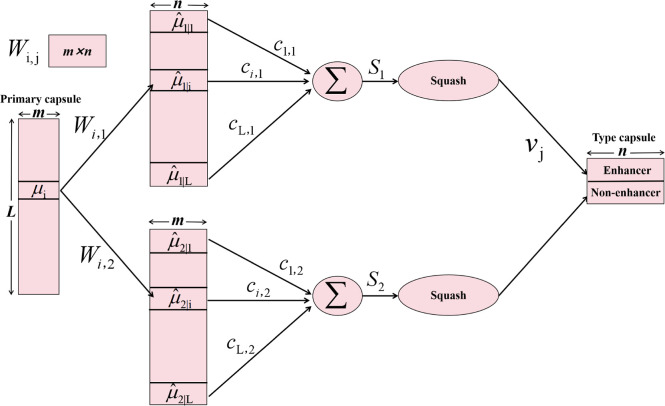
Computational process between primary capsules and type capsules.

For the binary classification task, the type capsule layer encompasses two *n*-dimensional capsules: one positive capsule and one negative capsule. The length of each capsule in type capsule layer represents the probability of being predicted as a positive (or negative) sample. Figure 4 illustrates the computational process between the primary and type capsule layers.

To derive the prediction vectors from capsule *i* to *j*, the outputs of the primary capsule layer *u*_*i*_ are initially multiplied by a learnable weight matrix *W*_*i*,*j*_. Subsequently, *S*_*j*_ is determined as the weighted sum of all computed .
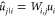
5
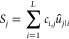
6where *i* and *j*, respectively, denote two capsules originating from the primary capsule layer and the type capsule layer, and *L* is the number of primary capsules. Here *c*_*i*,*j*_ represents coupling coefficients, determined by the dynamic routing algorithm (see Algorithm S1 in Supporting Information), indicating the degree of coupling between the primary capsule *i* and the type capsule *j*. *S*_*j*_ is fed into the Squash function to produce an output vector *V*_*j*_ with a length between 0 and 1.

Based on the theory of capsule networks, the vector *V*_*j*_ is utilized to model positive and negative samples, specifically (enhancer versus nonenhancer or strong enhancer versus weak enhancer) in this task. Each element of *V*_*j*_ represents a feature of positive or negative samples, and the length of *V*_*j*_ signifies the probability of being predicted as a positive or negative sample. Hence, to derive the predicted probabilities, it is necessary, at the network’s terminus, to compute the length of *V*_*j*_, as delineated by the following formula.
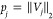
7where *p*_*j*,_ respectively, denote the model’s predictions for being in the positive class or negative class.

### Performance Assessment

In this study, we used accuracy, sensitivity, specificity, and the Matthews correlation coefficient (MCC) as evaluation metrics for the two-stage task. Their definitions are as follows.
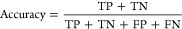
8
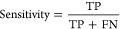
9
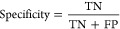
10
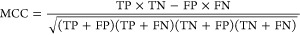
11where TP, TN, FP and FN represent the number of true positives, true negatives, false positives and false negatives, respectively.

The CGR encoding was implemented using the R package “Kaos”.^47^ The model was trained for 100 epochs to ensure adequate fitting, employing the Adam optimizer^48^ with an initial learning rate set to 0.1. Hyperparameter tuning was conducted using grid search and cross-validation techniques, with the specific search space outlined in Table S1. We iterate over all possible combinations of specified hyperparameter values and evaluate each combination using fivefold cross-validation to identify the best-performing hyperparameter combination. The pipeline for CapsEnhancer was established using PyTorch,^49^ and the training process utilized 4 × Nvidia 2080 Ti GPUs.

## Results and Discussion

### Performance Comparison with Existing Methods

#### First Stage: Enhancer Versus Nonenhancer

The capsule network-based with CGR encoding learning scheme was employed to adapt the task of standard binary classification between enhancer and nonenhancer sequences. We conducted a fair comparison with 13 currently existing tools, reporting performance on an independent test set as shown in Table 3. From Table 3, it is evident that CapsEnhancer achieves state-of-the-art performance compared to existing methods, exhibiting a substantial improvement in terms of accuracy, sensitivity, specificity, MCC, and AUC. In comparison to the second-ranked NEPERS method, CapsEnhancer exhibits an 8% improvement in ACC, reaching an accuracy of 94.5%, indicative of its precision in enhancer prediction. In terms of MCC, CapsEnhancer outperforms the second-ranked method by 0.16, reaching a value of 0.89. Furthermore, CapsEnhancer excels in sensitivity and specificity, surpassing the second-ranked method by 6 and 10%, reaching 93 and 96%, respectively. Notably, both sensitivity and specificity for CapsEnhancer exceed 90%, indicating its ability to provide more balanced predictions.

**Table 3:** Performance Comparison with Other Existing Methods on the Independent Test Set of the First Stage: Enhancer Versus Non-Enhancer

method	accuracy (%)	sensitivity (%)	specificity (%)	MCC	AUC (%)
iEnhancer-2L	73.0	71.0	75.0	0.460	80.6
EnhancerPred	74.0	73.5	74.5	0.480	80.1
iEnhancer-EL	74.8	71.0	78.5	0.496	81.7
iEnhancer-ECNN	76.9	78.5	75.2	0.537	83.2
iEnhancer-XG	75.8	74.0	77.5	0.515	
Enhancer-FRL	78.0	80.5	75.5	0.561	85.7
BERT-Enhancer	75.6	80.0	71.2	0.514	
iEnhancer-EBLSTM	77.2	75.5	79.5	0.534	83.5
iEnhancer-RF	79.8	78.5	81.0	0.595	86.0
iEnhancer-RD	78.8	81.0	76.5	0.576	84.4
spEnhancer	77.3	83.0	71.5	0.579	82.4
iEnhancer-DCSA	82.5	79.5	85.5	0.651	85.6
NEPERS	86.3	86.5	86.0	0.725	94.8
**CapsEnhancer (ours)**	**94.5**	**93.0**	**96.0**	**0.890**	**98.0**

Moreover, a significant challenge for two-stage tasks is how to handle false positive samples from the first stage. These false positives will also undergo the prediction task in the second stage, thereby affecting the robustness of the model. As shown in Table 3, it is evident that CapsEnhancer has a very low false positive rate (1—*specificity*) of only 4%, which is more than a 10% reduction compared to other existing methods. This demonstrates that CapsEnhancer is more robust than other methods and better at avoiding false positives.

#### Second Stage: Strong Enhancer Versus Weak Enhancer

The second stage of the CapsEnhancer involves the task of predicting enhancer strength, specifically distinguishing between strong enhancers and weak enhancers. We compared the performance of the second stage with existing methods, and the results are presented in Table 4. CapsEnhancer continues to exhibit impeccable predictive performance in the second stage, showcasing a significant lead in metrics such as accuracy, sensitivity, specificity, MCC, and AUC. In terms of accuracy, CapsEnhancer outperforms the second-ranked iEnhancer-DCSA by 3.5%, achieving a remarkable accuracy of 95%. Furthermore, in terms of MCC, it surpasses the second position by 0.06, reaching a value of 0.903. CapsEnhancer demonstrates satisfactory performance in sensitivity and specificity, achieving 99 and 91%, respectively.

**Table 4:** Performance Comparison with Other Existing Methods on the Independent Test Set of the Second Stage: Strong Enhancer Versus Weak Enhancer

method	accuracy (%)	sensitivity (%)	specificity (%)	MCC	AUC (%)
iEnhancer-2L	60.5	47.0	74.0	0.218	66.8
EnhancerPred	55.0	45.0	65.0	0.102	57.9
iEnhancer-EL	61.0	54.0	68.0	0.222	68.0
iEnhancer-ECNN	67.8	79.1	56.4	0.368	74.8
iEnhancer-XG	63.5	70.0	57.0	0.272	
Enhancer-FRL	73.5	98.0	49.0	0.539	87.2
BERT-Enhancer					
iEnhancer-EBLSTM	65.8	81.2	53.6	0.324	68.8
iEnhancer-RF	85.0	93.0	77.0	0.709	97.0
iEnhancer-RD	70.5	84.0	57.0	0.426	79.2
spEnhancer	62.0	91.0	33.0	0.370	62.5
iEnhancer-DCSA	91.5	98.0	85.0	0.837	96.6
NEPERS	89.0	94.0	84.0	0.784	95.1
**CapsEnhancer (ours)**	**95.0**	**99.0**	**91.0**	**0.903**	**99.2**

The results above demonstrate that CapsEnhancer has achieved outstanding performance in both stages of the task, which can be attributed to several factors. First, the use of CGR encoding serves as an efficient method for converting DNA sequences into two-dimensional images, enabling the application of computer vision techniques to sequence-related problems. Importantly, CGR encoding excels in capturing the frequency of Kmers.^35^ Prior literature has emphasized the significance of Kmers frequency as a critical feature in DNA sequence analysis.^46,50,51^

Second, owing to the architecture of the capsule network, the introduction of the capsule concept allows for effective spatial modeling of input images. Capsule networks overcome traditional CNN limitations, such as the inability to comprehend spatial relationships between features and the loss of invariance due to pooling operations. Consequently, in this context, capsule networks successfully learn the relationships between Kmers. Furthermore, owing to the aforementioned advantages of CapsEnhancer, we conducted a case study to illustrate its efficacy in managing sequencing errors and its capability to extend effectively to sequences of nonuniform lengths. Detailed information can be found in the case study section in Supporting Information. In conclusion, the synergistic combination of CGR encoding and capsule networks constitutes a pivotal factor in improving the performance of enhancer prediction tasks.

### Effectiveness of the Capsule Network Architecture

**Figure 5:**
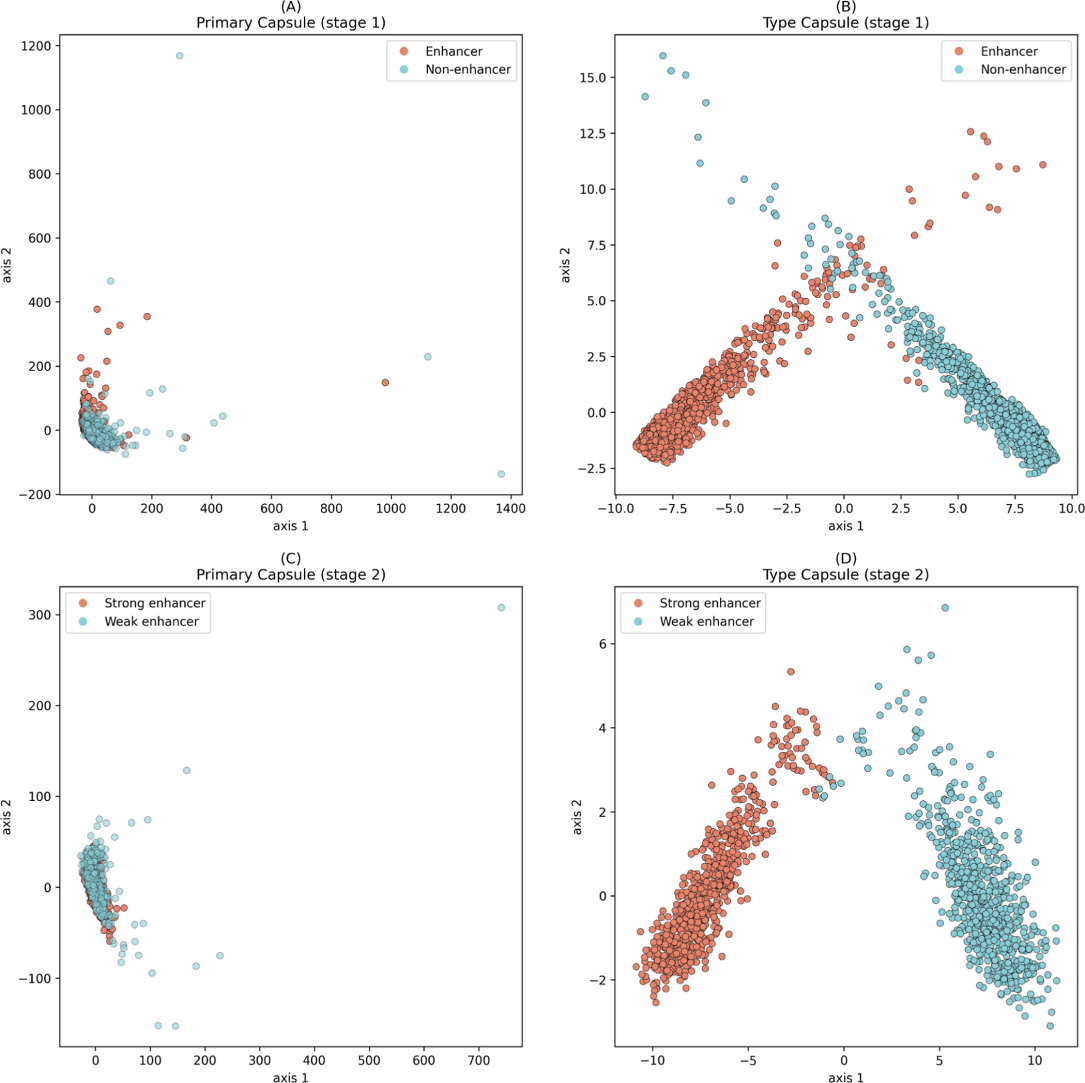
Visualization of positive and negative samples of train set in primary capsule and type capsule layers of CapsEnhancer in two stages. (A) Primary capsule layer of stage 1. (B) Type capsule layer of stage 1. (C) Primary capsule layer of stage 2. (D) Type capsule layer of stage 2.

The interaction between the primary capsule layer and the type capsule layer is at the core of the entire capsule network architecture. To visually demonstrate the superiority of the capsule network architecture, we extracted features from the samples in train sets at both the primary capsule layer and the class capsule layer in two distinct stages. Subsequently, utilizing the PCA dimensionality reduction technique, we reduced the extracted features to 2 dimensions, corresponding to the scatter plots shown in Figure 5. Red and blue points represent positive and negative samples, respectively (stage 1: enhancer and nonenhancer; stage 2: strong enhancer and weak enhancer).

From Figure 5A,C, it can be observed that the points representing positive and negative samples are entangled, exhibiting similar distributions, making it challenging to distinguish between them. However, after undergoing the capsule network architecture, the red and blue points manifest clearly distinct distributions, facilitating easy differentiation. This indicates that the computational processes in the primary capsule layer and class capsule layer further refine the features. The processed features enable positive and negative samples to exhibit disparate distributions, thereby enhancing the predictive capabilities of the model.

This improvement is attributed to the dynamic routing algorithm of the capsule network, which allows information propagation and weight adjustments between different capsules. This dynamic routing mechanism is crucial for the capsule network’s ability to model spatial relationships among different features.

### Ablation Experiment

Subsequently, we conducted ablation experiments to further validate the significance of the capsule network. We replaced the capsule network with a multilayer perceptron and performed experiments at both stages. The experimental results are presented in Table 5. It is evident that upon removing the capsule network, the model’s performance significantly deteriorated at both stages. In the first stage, the accuracy dropped by 17%, reaching only 77.3% compared to CapsEnhancer. In the second stage, the accuracy was lower by 9%, reaching only 86% compared to CapsEnhancer. In terms of MCC, the absence of the capsule network architecture resulted in a decrease of 0.345 and 0.161 at the two stages, achieving MCC values of 0.545 and 0.742, respectively.

**Table 5:** Performance Comparison with Other Existing Methods on the Independent Test Set of the Second Stage: Strong Enhancer Versus Weak Enhancer

stage	method	accuracy (%)	sensitivity (%)	specificity (%)	MCC	AUC (%)
first stage	without CapsNet	77.3	79.0	75.5	0.55	82.8
	CapsEnhancer	94.5	93.0	96.0	0.80	98.0
second stage	without CapsNet	86.0	98.0	74.0	0.72	91.8
	CapsEnhancer	95.0	99.0	91.0	0.93	99.2

Furthermore, in the second stage of the model, without the capsule network, the sensitivity and specificity were 98 and 74%, respectively. This indicates that in the absence of the capsule network, the model not only fails to achieve precise predictions but also lacks the ability to achieve a balanced prediction.

**Figure 6:**
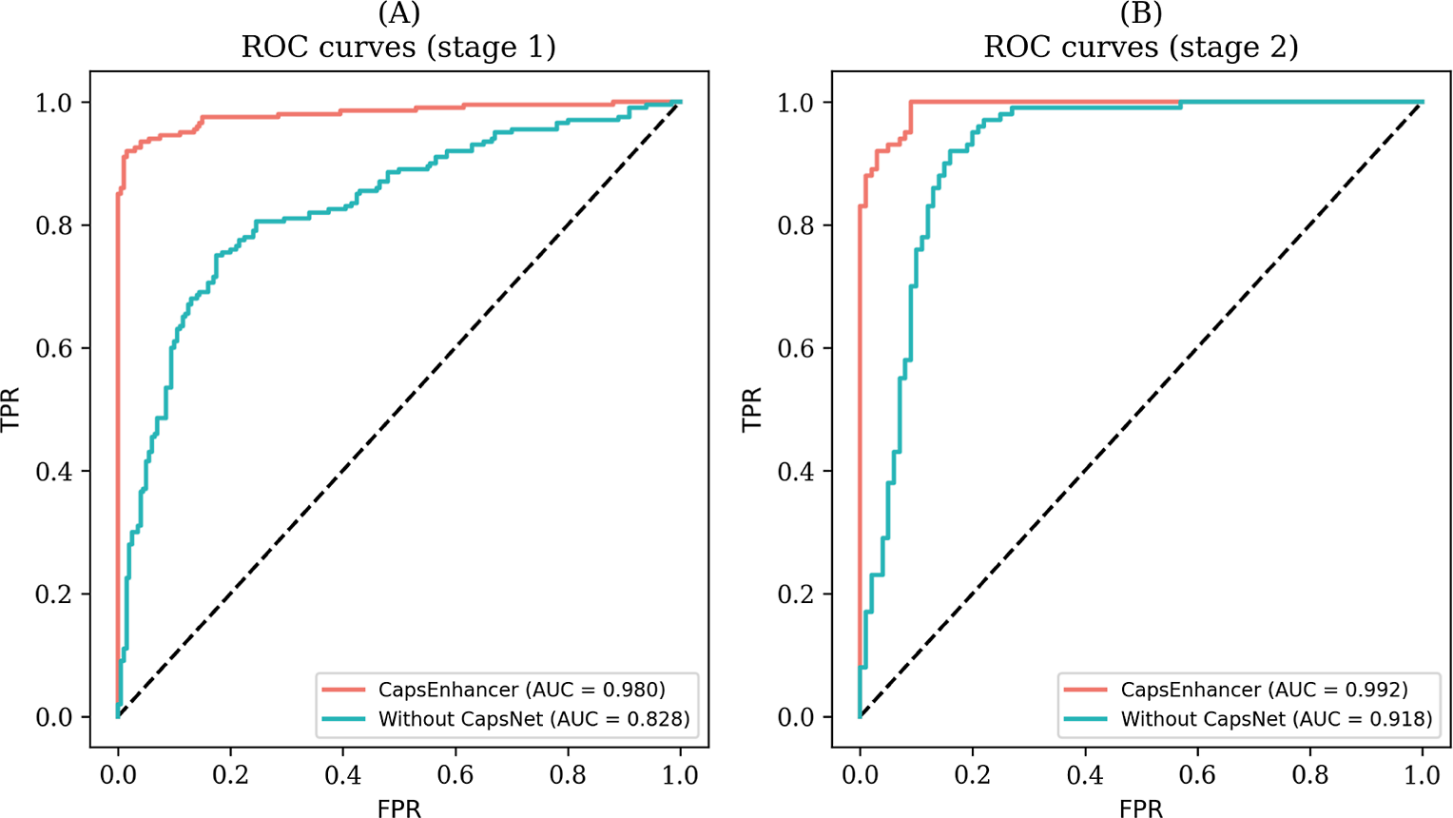
ROC curves for CapsEnhancer and the model without capsule network in (A) stage 1 and (B) stage 2.

In order to visually demonstrate the performance of the ablation experiments, we plotted the receiver operating characteristic (ROC) curves for CapsEnhancer and models without CapsNst, in two stages as illustrated in Figure 6. As evident from Figure 6, whether in stage 1 or stage 2, the ROC curve corresponding to CapsEnhancer consistently resides outside that of the model without CapsNst, achieving a higher AUC. In addition, we also plotted the precision-recall (PR) curves for both stages, as depicted in Figure S3. Similar to ROC curves, CapsEnhancer achieved a higher area under the PR compared to models without CapsNet. This further underscores the significance of the capsule network architecture in improving the predictive capabilities for the enhancer task.

### Feature Analysis

**Figure 7:**
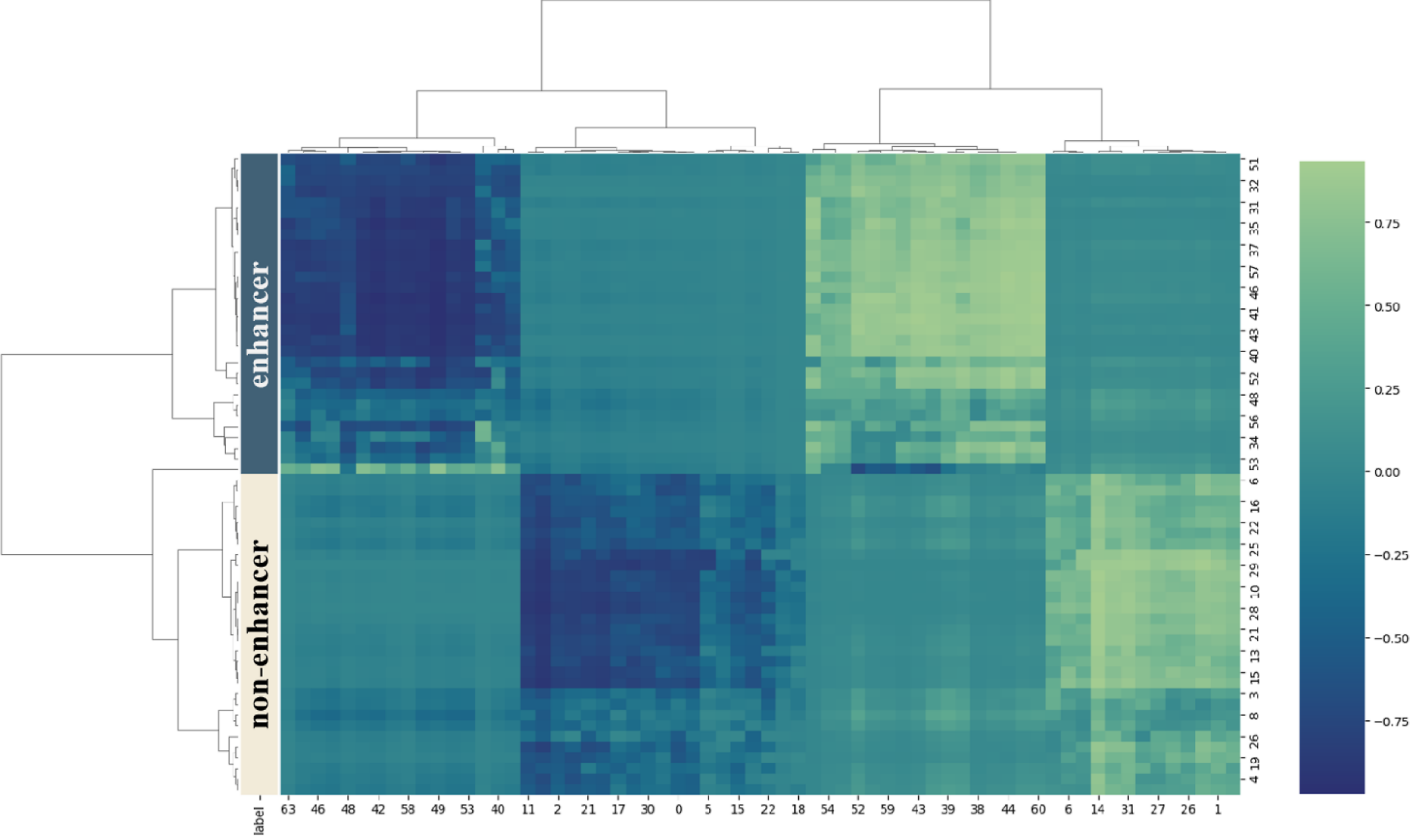
Clustering analysis map of latent features generated by CapsEnhancer on the independent test set in stage 1.

Within the domain of deep learning, discriminative features play a pivotal role in the development of robust classifiers. In contrast to existing methods, CapsEnhancer exhibits dual principal advantages: first, it leverages CGR encoding for the representation of DNA sequences, and second, it effectively learns from CGR images through the deployment of capsule network architecture. Consider the first stage, where the type capsule layer encompasses two capsules, each constituting a 32-dimensional vector corresponding to enhancer or nonenhancer categories. This configuration facilitates the construction of distinct features associated with enhancer and nonenhancer attributes. To underscore the discriminative efficacy of features extracted by CapsEnhancer concerning enhancers, 30 enhancers and 30 nonenhancers were randomly selected from the test set for comprehensive feature clustering analysis on the corresponding DNA sequences. In this analysis, we used hierarchical clustering with the complete linkage method. The resultant clustering patterns, illustrated in Figure 7, reveal two key observations: enhancers and nonenhancers distinctly cluster into separate subtrees, and DNA sequences of the same classification often exhibit analogous feature patterns. These findings provide compelling evidence that the features derived from the proposed CapsEnhancer method adeptly encapsulate traits pertinent to enhancers, offering further justification for the method’s effectiveness.

## Conclusions

Enhancers are a type of noncoding DNA element that can regulate gene expression. The identification of enhancers is crucial in the field of biology. First, it provides insights into the complex networks of gene regulation that govern various biological processes, such as development, differentiation, and response to environmental stimuli. By pinpointing enhancers associated with specific genes, researchers can unravel the molecular mechanisms underlying normal cellular functions and pathological conditions. Furthermore, the identification of enhancers has significant implications in the context of human health. Dysregulation of gene expression, often influenced by aberrant enhancer activity, is implicated in numerous diseases, including cancers and developmental disorders. Unraveling enhancer landscapes helps researchers identify potential therapeutic targets and develop strategies for precise intervention in gene expression patterns.

Traditional experimental methods, while effective in identifying enhancers, often demand substantial human and financial resources. In recent years, there has been an increasing emphasis on employing computational approaches for enhancer identification, driven by the rapid advancements in artificial intelligence methods. In this study, we propose a two-stage framework, CapsEnhancer, based on deep learning to efficiently predict enhancers and their strengths. The first stage focuses on identifying enhancers, while the second stage aims to predict strong and weak enhancers. Initially, we employ CGR encoding to represent each DNA sequence as an image, enabling the efficient representation of Kmers and frequencies. Furthermore, we utilize a capsule network-based architecture to extract local and global features of the images, overcoming the limitations of traditional CNNs and providing spatial modeling for features of these images. Experimental results demonstrate the outstanding predictive capabilities of our method in both stages, achieving state-of-the-art performance. This study employs computer vision methods to handle sequence data, and we believe that our research not only offers novel insights into enhancer identification but also provides a fresh perspective for other biological sequence analysis tasks.

## Key Points

- We proposed a two-stage framework, CapsEnhancer, based on deep learning, for accurate prediction of enhancers and their strength.
- CapsEnhancer employs CGR encoding to represent each DNA sequence as an image. Through this encoding methodology, it enables effective representation of Kmers and their frequencies.
- CapsEnhancer utilizes an architecture based on capsule networks to learn both local and global features from DNA “images”. Capsule networks overcome the limitations of traditional CNNs by capturing spatial relationships among features in DNA “images”, thereby enhancing the model’s performance.
- The framework proposed in our study employs computer vision strategies to process biosequence data, complemented by the integration of a next-generation neural network, the capsule network. This presents a novel approach and perspective for tasks of biosequence data analysis.

## Data Availability

Availability and implementationCapsEnhancer and data sets of this study are available at https://github.com/Cpillar/CapsEnhancer.

## Supporting Information Available

GC content distribution of the data sets, FCGR images for two example sequences and PR curves for the ablation experiment, hyperparameters of CapsEnhancer, a case study, and description of the dynamic routing algorithm (PDF)

## References

[ref1] Basith S.; Hasan M. M.; Lee G.; Wei L.; Manavalan B. Integrative machine learning framework for the identification of cell-specific enhancers from the human genome. Briefings Bioinf. 2021, 22, bbab252 10.1093/bib/bbab252. 34226917

[ref2] Corradin O.; Scacheri P. Enhancer variants: evaluating functions in common disease. Genome Med. 2014, 6 (10), 85 10.1186/s13073-014-0085-3. 25473424 PMC4254432

[ref3] Levine M. Transcriptional enhancers in animal development and evolution. Curr. Biol. 2010, 20, R754–R763. 10.1016/j.cub.2010.06.070. 20833320 PMC4280268

[ref4] Zhang L.; Yang Y.; Chai L.; Li Q.; Liu J.; Lin H.; Liu L. A deep learning model to identify gene expression level using cobinding transcription factor signals. Briefings Bioinf. 2022, 23, bbab501 10.1093/bib/bbab501. 34864886

[ref5] Heinz S.; Romanoski C. E.; Benner C.; Glass C. K. The selection and function of cell type-specific enhancers. Nat. Rev. Mol. Cell Biol. 2015, 16, 144–154. 10.1038/nrm3949. 25650801 PMC4517609

[ref6] Furlong E. E.; Levine M. Developmental enhancers and chromosome topology. Science 2018, 361, 1341–1345. 10.1126/science.aau0320. 30262496 PMC6986801

[ref7] Schoenfelder S.; Fraser P. Long-range enhancer–promoter contacts in gene expression control. Nat. Rev. Genet. 2019, 20, 437–455. 10.1038/s41576-019-0128-0. 31086298

[ref8] Bauer D. E.; Orkin S. H. Hemoglobin switching’s surprise: the versatile transcription factor BCL11A is a master repressor of fetal hemoglobin. Curr. Opin. Genet. Dev. 2015, 33, 62–70. 10.1016/j.gde.2015.08.001. 26375765 PMC4705561

[ref9] Chen X.; Xu H.; Yuan P.; Fang F.; Huss M.; Vega V. B.; Wong E.; Orlov Y. L.; Zhang W.; Jiang J.; et al. Integration of external signaling pathways with the core transcriptional network in embryonic stem cells. Cell 2008, 133, 1106–1117. 10.1016/j.cell.2008.04.043. 18555785

[ref10] May D.; Blow M. J.; Kaplan T.; McCulley D. J.; Jensen B. C.; Akiyama J. A.; Holt A.; Plajzer-Frick I.; Shoukry M.; Wright C.; et al. Large-scale discovery of enhancers from human heart tissue. Nat. Genet. 2012, 44, 89–93. 10.1038/ng.1006. PMC3246570 22138689

[ref11] Visel A.; Blow M. J.; Li Z.; Zhang T.; Akiyama J. A.; Holt A.; Plajzer-Frick I.; Shoukry M.; Wright C.; Chen F.; et al. ChIP-seq accurately predicts tissue-specific activity of enhancers. Nature 2009, 457, 854–858. 10.1038/nature07730. 19212405 PMC2745234

[ref12] Pennacchio L. A.; Bickmore W.; Dean A.; Nobrega M. A.; Bejerano G. Enhancers: five essential questions. Nat. Rev. Genet. 2013, 14, 288–295. 10.1038/nrg3458. 23503198 PMC4445073

[ref13] Ku C. S.; Naidoo N.; Wu M.; Soong R. Studying the epigenome using next generation sequencing. J. Med. Genet. 2011, 48, 721–730. 10.1136/jmedgenet-2011-100242. 21825079

[ref14] Liu B.; Fang L.; Long R.; Lan X.; Chou K.-C. iEnhancer-2L: a two-layer predictor for identifying enhancers and their strength by pseudo k-tuple nucleotide composition. Bioinformatics 2016, 32, 362–369. 10.1093/bioinformatics/btv604. 26476782

[ref15] Jia C.; He W. EnhancerPred: a predictor for discovering enhancers based on the combination and selection of multiple features. Sci. Rep. 2016, 6, 38741 10.1038/srep38741. 27941893 PMC5150536

[ref16] Lim D. Y.; Khanal J.; Tayara H.; Chong K. T. iEnhancer-RF: identifying enhancers and their strength by enhanced feature representation using random forest. Chemom. Intell. Lab. Syst. 2021, 212, 104284 10.1016/j.chemolab.2021.104284.

[ref17] Liu B.; Li K.; Huang D.-S.; Chou K.-C. iEnhancer-EL identifying enhancers and their strength with ensemble learning approach. Bioinformatics 2018, 34, 3835–3842. 10.1093/bioinformatics/bty458. 29878118

[ref18] Wang C.; Zou Q.; Ju Y.; Shi H. Enhancer-FRL: improved and robust identification of enhancers and their activities using feature representation learning. IEEE/ACM Trans. Comput. Biol. Bioinform. 2023, 20, 967–975. 10.1109/TCBB.2022.3204365. 36063523

[ref19] Gill M.; Ahmed S.; Kabir M.; Hayat M. A novel predictor for the analysis and prediction of enhancers and their strength via multi-view features and deep forest. Information 2023, 14, 636 10.3390/info14120636.

[ref20] Nguyen Q. H.; Nguyen-Vo T.-H.; Le N. Q. K.; Do T. T.; Rahardja S.; Nguyen B. P. iEnhancer-ECNN: identifying enhancers and their strength using ensembles of convolutional neural networks. BMC Genom. 2019, 20, 951 10.1186/s12864-019-6336-3. PMC6929481 31874637

[ref21] Le N. Q. K.; Ho Q.-T.; Nguyen T.-T.-D.; Ou Y.-Y. A transformer architecture based on BERT and 2D convolutional neural network to identify DNA enhancers from sequence information. Briefings Bioinf. 2021, 22, bbab005 10.1093/bib/bbab005. 33539511

[ref22] Niu K.; Luo X.; Zhang S.; Teng Z.; Zhang T.; Zhao Y. iEnhancer-EBLSTM: identifying enhancers and strengths by ensembles of bidirectional long short-term memory. Front. Genet. 2021, 12, 665498 10.3389/fgene.2021.665498. 33833783 PMC8021722

[ref23] Cai L.; Ren X.; Fu X.; Peng L.; Gao M.; Zeng X. iEnhancer-XG: interpretable sequence-based enhancers and their strength predictor. Bioinformatics 2021, 37, 1060–1067. 10.1093/bioinformatics/btaa914. 33119044

[ref24] Yang H.; Wang S.; Xia X. iEnhancer-RD: identification of enhancers and their strength using RKPK features and deep neural networks. Anal. Biochem. 2021, 630, 114318 10.1016/j.ab.2021.114318. 34364858

[ref25] Mu X.; Wang Y.; Duan M.; Liu S.; Li F.; Wang X.; Zhang K.; Huang L.; Zhou F. A novel position-specific encoding algorithm (SeqPose) of nucleotide sequences and its application for detecting enhancers. Int. J. Mol. Sci. 2021, 22, 3079 10.3390/ijms22063079. 33802922 PMC8002641

[ref26] Luo H.; Chen C.; Shan W.; Ding P.; Luo L. iEnhancer-BERT: a novel transfer learning architecture based on DNA-Language model for identifying enhancers and their strength. In International Conference on Intelligent Computing, 2022; pp 153–165. 10.1007/978-3-031-13829-4_13.

[ref27] Li J.; Wu Z.; Lin W.; Luo J.; Zhang J.; Chen Q.; Chen J. iEnhancer-ELM: improve enhancer identification by extracting position-related multiscale contextual information based on enhancer language models. Bioinform. Adv. 2023, 3, vbad043 10.1093/bioadv/vbad043. 37113248 PMC10125906

[ref28] Wang W.; Wu Q.; Li C. iEnhancer-DCSA: identifying enhancers via dual-scale convolution and spatial attention. BMC Genom. 2023, 24, 393 10.1186/s12864-023-09468-1. PMC10339552 37442977

[ref29] Wu H.; Liu M.; Zhang P.; Zhang H. iEnhancer-SKNN: a stacking ensemble learning-based method for enhancer identification and classification using sequence information. Briefings Funct. Genomics 2023, 22, 302–311. 10.1093/bfgp/elac057. 36715222

[ref30] Ng P. dna2vec: consistent vector representations of variable-length k-mers. arXiv 2017, arXiv:1701.06279.

[ref31] Iuchi H.; Matsutani T.; Yamada K.; Iwano N.; Sumi S.; Hosoda S.; Zhao S.; Fukunaga T.; Hamada M. Representation learning applications in biological sequence analysis. Comput. Struct. Biotechnol. J. 2021, 19, 3198–3208. 10.1016/j.csbj.2021.05.039. 34141139 PMC8190442

[ref32] Wen J.; Liu Y.; Shi Y.; Huang H.; Deng B.; Xiao X. A classification model for lncRNA and mRNA based on k-mers and a convolutional neural network. BMC Bioinf. 2019, 20, 469 10.1186/s12859-019-3039-3. PMC6743109 31519146

[ref33] Li W.; Guo Y.; Wang B.; Yang B. Learning spatiotemporal embedding with gated convolutional recurrent networks for translation initiation site prediction. Pattern Recogn. 2023, 136, 109234 10.1016/j.patcog.2022.109234.

[ref34] Jeffrey H. J. Chaos game representation of gene structure. Nucleic Acids Res. 1990, 18, 2163–2170. 10.1093/nar/18.8.2163. 2336393 PMC330698

[ref35] Löchel H. F.; Eger D.; Sperlea T.; Heider D. Deep learning on chaos game representation for proteins. Bioinformatics 2020, 36, 272–279. 10.1093/bioinformatics/btz493. 31225868

[ref36] LaLonde R.; Bagci U. Capsules for object segmentation. arXiv 2018, arXiv:1804.04241.

[ref37] Dong Z.; Lin S. Research on image classification based on capsnet. In 2019 IEEE 4th Advanced Information Technology, Electronic and Automation Control Conference (IAEAC), 2019; pp 1023–1026.

[ref38] Guo Y.; Zhou D.; Ruan X.; Cao J. Variational gated autoencoder-based feature extraction model for inferring disease-miRNA associations based on multiview features. Neural Network. 2023, 165, 491–505. 10.1016/j.neunet.2023.05.052. 37336034

[ref39] Guo Y.; Zhou D.; Li P.; Li C.; Cao J. Context-aware poly (a) signal prediction model via deep spatial–temporal neural networks. IEEE Trans. Neural Netw. Learn. Syst. 2024, 35, 8241–8253. 10.1109/tnnls.2022.3226301. 37015693

[ref40] Wang X.; Guan Z.; Qian W.; Cao J.; Wang C.; Ma R. STFuse: infrared and visible image fusion via semisupervised transfer learning. IEEE Trans. Neural Netw. Learn. Syst. 2024, 1–14. 10.1109/tnnls.2023.3328060. 37938956

[ref41] Sabour S.; Frosst N.; Hinton G. E. Dynamic routing between capsules. In Advances in Neural Information Processing Systems, 2017; Vol. 30.

[ref42] Yao L.; Pang Y.; Wan J.; Chung C.-R.; Yu J.; Guan J.; Leung C.; Chiang Y.-C.; Lee T.-Y. ABPCaps: a novel capsule network-based method for the prediction of antibacterial peptides. Appl. Sci. 2023, 13, 6965 10.3390/app13126965.

[ref43] Huang Y.; Huang H.-Y.; Chen Y.; Lin Y.-C.-D.; Yao L.; Lin T.; Leng J.; Chang Y.; Zhang Y.; Zhu Z.; et al. A robust drug–target interaction prediction framework with capsule network and transfer learning. Int. J. Mol. Sci. 2023, 24, 14061 10.3390/ijms241814061. 37762364 PMC10531393

[ref44] Wang D.; Liang Y.; Xu D. Capsule network for protein post-translational modification site prediction. Bioinformatics 2019, 35, 2386–2394. 10.1093/bioinformatics/bty977. 30520972 PMC6612812

[ref45] Khanal J.; Tayara H.; Zou Q.; To Chong K. DeepCap-Kcr: accurate identification and investigation of protein lysine crotonylation sites based on capsule network. Briefings Bioinf. 2022, 23, bbab492 10.1093/bib/bbab492. 34882222

[ref46] Shang J.; Peng C.; Tang X.; Sun Y. PhaVIP: Phage VIrion protein classification based on chaos game representation and vision transformer. arXiv 2023, arXiv:2301.12422. 10.1093/bioinformatics/btad229 PMC10311294 37387136

[ref47] Löchel H. F.; Heider D. Chaos game representation and its applications in bioinformatics. Comput. Struct. Biotechnol. J. 2021, 19, 6263–6271. 10.1016/j.csbj.2021.11.008. 34900136 PMC8636998

[ref48] Kingma D. P.; Ba J. Adam: a method for stochastic optimization. arXiv 2014, arXiv:1412.6980.

[ref49] Paszke A.; Gross S.; Massa F.; Lerer A.; Bradbury J.; Chanan G.; Killeen T.; Lin Z.; Gimelshein N.; Antiga L.; Pytorch: an imperative style, high-performance deep learning library. In Advances in Neural Information Processing Systems, 2019; Vol. 32.

[ref50] Kishk A.; Elzizy A.; Galal D.; Razek E. A.; Fawzy E.; Ahmed G.; Gawish M.; Hamad S.; El-Hadidi M. A hybrid machine learning approach for the phenotypic classification of metagenomic colon cancer reads based on kmer frequency and biomarker profiling. In 2018 9th Cairo International Biomedical Engineering Conference (CIBEC), 2018; pp 118–121.

[ref51] Yin B.; Balvert M.; Zambrano D.; Schönhuth A.; Bohte S. An image representation based convolutional network for DNA classification. arXiv 2018, arXiv:1806.04931.

