# Supplementary material for: CapsEnhancer: An Effective Computational Framework for Identifying Enhancers Based on Chaos Game Representation and Capsule Network
Source: J Chem Inf Model. 2024 Jul 1;64(14):5725–36. doi: 10.1021/acs.jcim.4c00546 (PMC11267569; doi:10.1021/acs.jcim.4c00546)
Supplement: Supplementary file 1 — ci4c00546_si_001.pdf [file ci4c00546_si_001.pdf]

Supporting Information

CapsEnhancer: an effective computational framework for identifying enhancers based on chaos game representation and capsule network

Lantian Yao,[1,2,†] Peilin Xie,[1,†] Jiahui Guan,[3] Chia-Ru Chung,[4] Yixian Huang,[3] Yuxuan Pang,[5] Huacong Wu,[3] Ying-Chih Chiang[*,1,3] and Tzong-Yi Lee[*,6,7]

1. Kobilka Institute of Innovative Drug Discovery, School of Medicine, The Chinese University of Hong Kong, Shenzhen, China, 518172
2. School of Science and Engineering, The Chinese University of Hong Kong, Shenzhen, China, 518172
3. School of Medicine, The Chinese University of Hong Kong, Shenzhen, China, 518172
4. Department of Computer Science and Information Engineering, National Central University, Taoyuan, Taiwan, 320317
5. Division of Health Medical Intelligence, Human Genome Center, The Institute of Medical Science, The University of Tokyo, Tokyo, Japan, 108-8639
6. Institute of Bioinformatics and Systems Biology, National Yang Ming Chiao Tung University, Hsinchu, Taiwan, 300093
7. Center for Intelligent Drug Systems and Smart Bio-devices (IDS2B), National Yang Ming Chiao Tung University, Hsinchu, 300, Taiwan, 300093

[†]These authors contributed equally to this work.
[*]Corresponding author. Tzong-Yi Lee: leetzongyi@nycu.edu.tw and Ying-Chih Chiang: chiangyc@cuhk.edu.cn

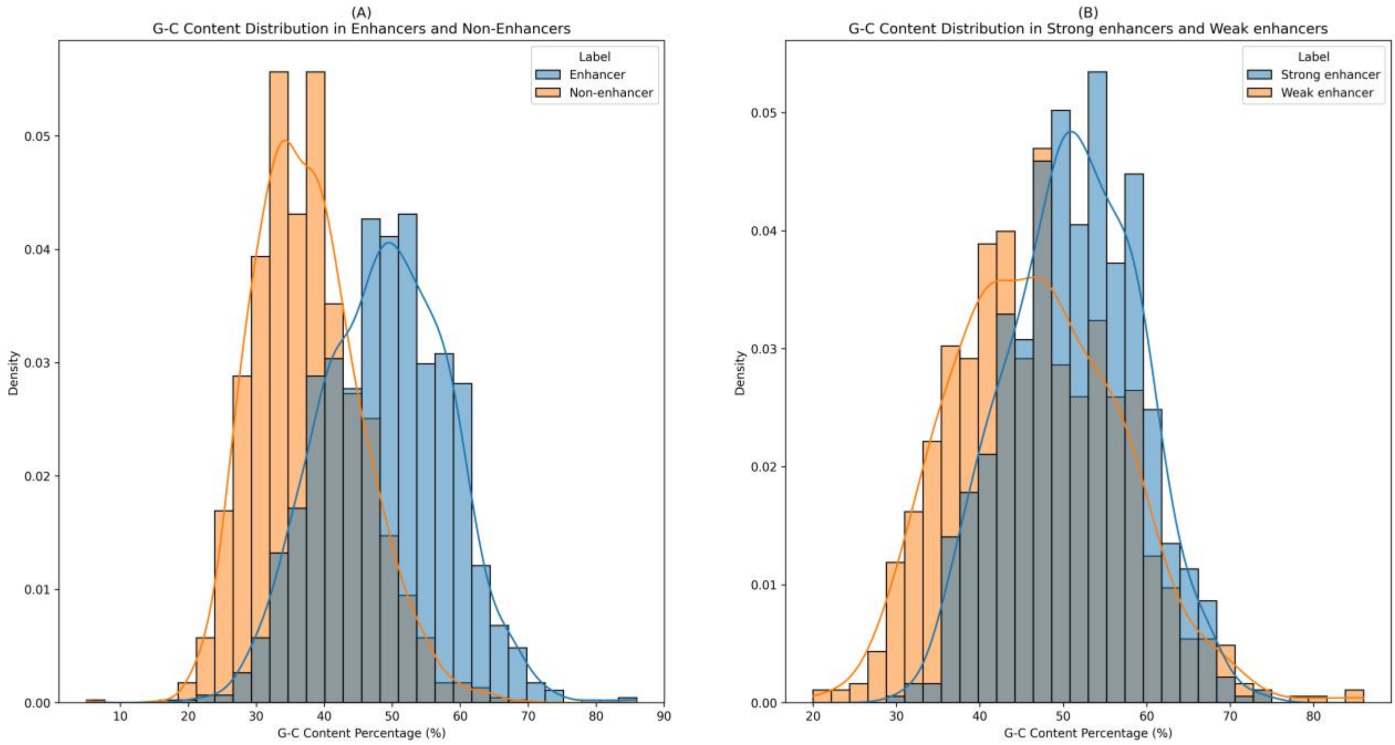

Figure S1. The GC content distribution for the (A) first stage and (B) second stage.

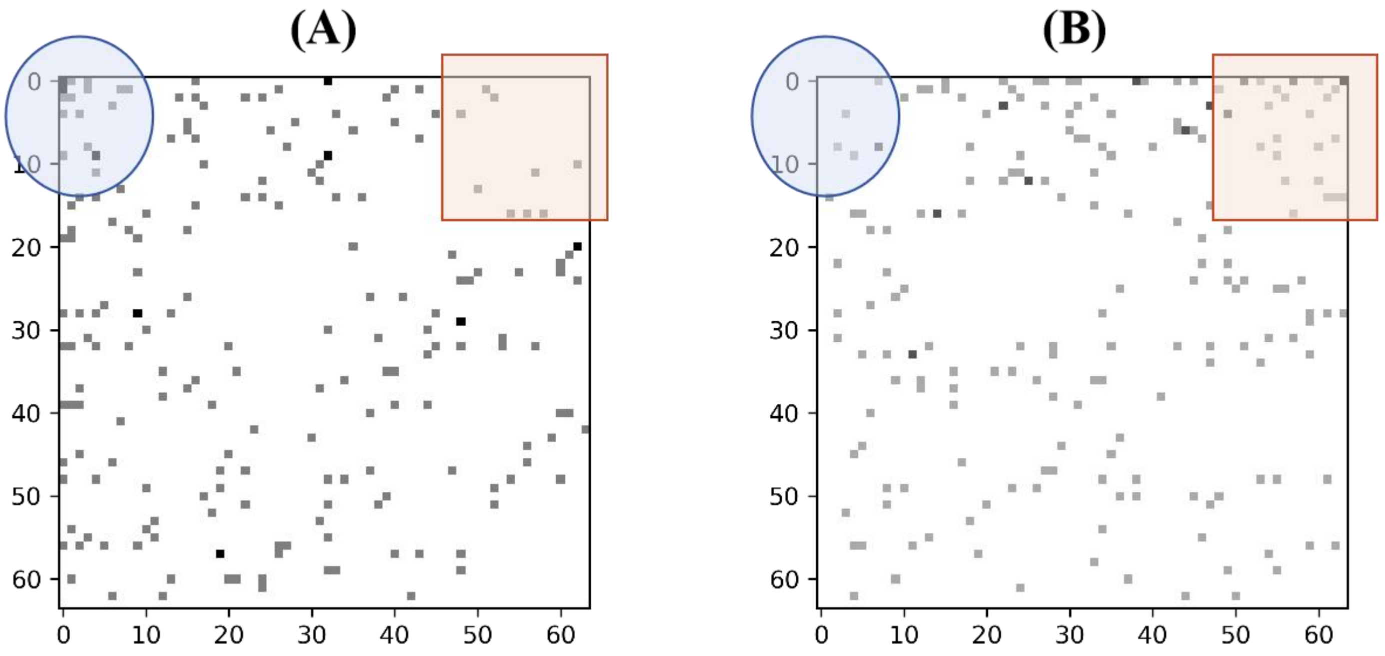

Figure S2. FCGR images for two example sequences. (A) enhancer (chrX_48897056_48897256). (B) non-enhancer (chr8_137170818_137171018). The regions marked in red and blue demonstrate different patterns.

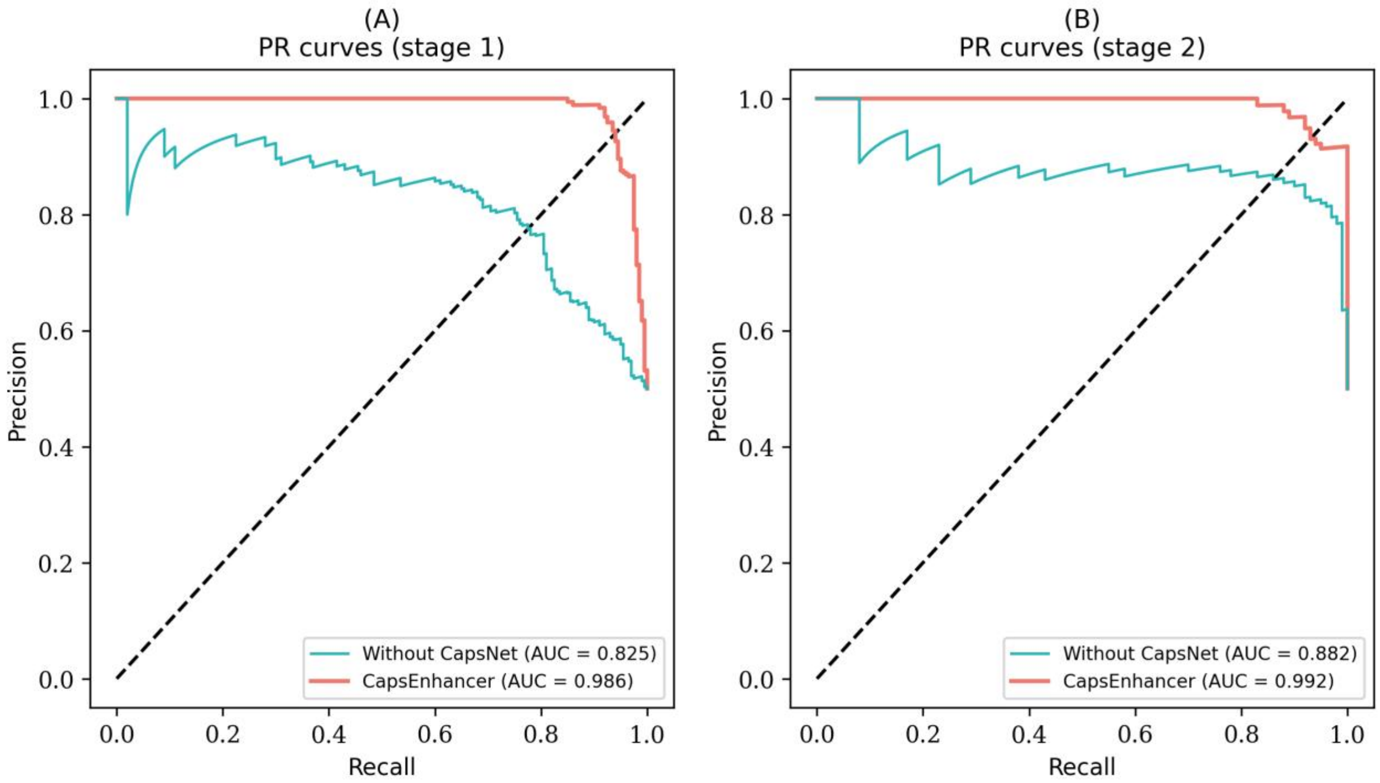

Figure S3. PR curves for CapsEnhancer and the model without capsule network in (A) stage 1 and (B) stage 2.

Table S1. The hyperparameters of CapsEnhancer.

Hyperparameters	Search space	Best hyperparameter (Stage 1)	Best hyperparameter (Stage 2)
The size of the FCGR images	{16, 32, 64, 128}	64	64
The kernel size of convolutional networks	{3, 5, 7, 9}	9	9
The number of primary capsules	{4, 8, 16, 32}	8	4
The number of routing iterations	{2, 3, 4}	2	2
The dimension of type capsules	{8, 16, 32}	32	32

Case study

In the real world, sequencing errors frequently occur. We designed a case study to demonstrate that CapsEnhancer can effectively handle sequencing errors and can be efficiently extended to sequences of non-uniform length.

We conducted experiments using enhancer data consistently present in four different databases: GeneHancer, dbSUPER, HEDD, and EnhancerAtlas (labeled as "1"), with the aim of enhancing the reliability of our data. The table below summarizes the results of the case study.

For sequencing errors, we utilized Single Nucleotide Variations (SNPs) to simulate the occurrence of sequencing errors. SNPs refer to the DNA sequence polymorphism caused by the variation of a single nucleotide at the genome level, which is conceptually similar to sequence errors. For instance, in the cerebellum tissue on chromosome 1, our model accurately predicted that position 2255646 would remain an enhancer, regardless of whether the nucleotide T mutated to A, C, or G. Results from our case study demonstrate that CapsEnhancer can effectively handle sequencing errors. This robustness stems from the use of FCGR encoding in our computational vision classifier, unlike typical models based on natural language processing (NLP). Minor sequence errors do not significantly affect the FCGR encoding, thus ensuring CapsEnhancer's resilience.

This case study also demonstrates CapsEnhancer's performance on sequences of varying lengths. In the study, we evaluated 12 enhancer sequences of different lengths (from above four databases) and accurately predicted 11 sequences, achieving an accuracy rate of 91.7%. These results demonstrate that CapsEnhancer performs exceptionally well across different sequence lengths. The reason lies in the FCGR encoding technique, which differs from traditional models that process sequences directly. Unlike NLP tasks that require padding or other modifications for different lengths, FCGR encoding can transform sequences of any length into a consistent format of images. This allows CapsEnhancer to accommodate and effectively process sequences of various lengths without additional adjustments.

The summary of the case study.

Organism	Tissue/cell	Coordinate	Length	SNP Status	Predict
Homo sapiens	A549	chr1:8058830-8067470	8640	Original sequence	1
				SNP (chr1:8005885; G>T)	1
Homo sapiens	Cerebellum	chr1:2186680-2189090	2410	Original sequence	1
				SNP (chr1:2255646; T>A)	1
				SNP (chr1:2255646; T>C)	1
				SNP (chr1:2255646; T>G)	1
Homo sapiens	CD19+	chr1:1705570-1709720	4150	Original sequence	0
				SNP (chr1:1774506; T>A)	0
				SNP (chr1:1774506; T>C)	0
				SNP (chr1:1774506; T>G)	0
Homo sapiens	CD19+	chr1:2185400-2189010	3610	Original sequence	1
				SNP (chr1:2255646; T>A)	1
				SNP (chr1:2255646;T>C)	1
				SNP (chr1:2255646; T>G)	1
Homo sapiens	NKC	chr1:16274990-16279110	4120	Original sequence	1
				SNP (chr1:15950588; A>C)	1

				SNP (chr1:15950589; A>G)	1
				Original sequence	1
				SNP (chr1:19514680; G>A)	1
Homo sapiens	Fetal_lung	chr1:19839100-19842170	3070	SNP (chr1:19512621;A>C)	1
				SNP(chr1:19512621;A>C) AND SNP(chr1:19514680;G>A)	1
				Original sequence	1
Homo sapiens	HACAT	chr1:16510810-16511820	1010	SNP (chr1:16184399;C>T)	1
				SNP (chr1:16184399;C>G)	1
				Original sequence	1
Homo sapiens	H2171	chr1:22538600-22540620	2020	SNP (chr1:22212294; C>T)	1
				SNP (chr1:22212294; C>G)	1
Homo sapiens	Hela	chr1:45011220-45013010	1790	Original sequence	1
				SNP (chr1:44546601; A>C)	1
Homo sapiens	NT2-D1	chr1:44010840-44012750	1910	Original sequence	1
				SNP (chr1:43546066; G>A)	1
				Original sequence	1
Homo sapiens	Retina	chr1:1252950-1254670	1720	SNP (chr1:1319056; A>T)	1
				SNP (chr1:1319056; A>C)	1
				SNP (chr1:1319056; A>G)	1
Homo sapiens	GM12891	chr1:957840-960340	2500	Original sequence	1
				SNP (chr1:1023573; A>G)	1

Algorithm 1: Dynamic Routing

Input: $\hat{\boldsymbol{u}}_{j|i}$ and $\boldsymbol{r}$

Output: $\boldsymbol{v_j}$

1. For all capsule $\boldsymbol{i}$ in primary capsule layer capsule $\boldsymbol{j}$ in type capsule layer: $\boldsymbol{b_{ij}} \leftarrow \boldsymbol{0}$
2. for $\boldsymbol{r}$ iterations do:
3. $\quad \boldsymbol{c_{ij}} \leftarrow \boldsymbol{softmax}(\boldsymbol{b_{ij}})$
4. $\quad \boldsymbol{S_j} \leftarrow \sum_i \boldsymbol{c_{ij}} \hat{\boldsymbol{u}}_{j|i}$
5. $\quad \boldsymbol{v_j} \leftarrow \boldsymbol{Squash}(\boldsymbol{S_j})$
6. $\quad \boldsymbol{b_{ij}} \leftarrow \boldsymbol{b_{ij}} + \hat{\boldsymbol{u}}_{j|i}\boldsymbol{v_j}$
7. end for
8. return $\boldsymbol{v_j}$

The comprehensive process of dynamic routing is depicted in Algorithm 1. The scalar b_{ij} serves as the log prior probability between the primary capsule i and the type capsule j, with c_{ij} being computed via a softmax function applied to b_{ij}. Consequently, the sum of the coupling coefficients from the primary capsule i to the type capsules, $\sum_{k=1}^{N} c_{ik}$, equals 1, where N stands for the number of type capsules. The iteration number r is a hyperparameter that is predetermined. During dynamic routing, the type capsule layer generates output vectors v_j. The elements within v_j encode the features, and their length indicates the probability distribution between the two types, enhancer and non-enhancer.
